# Civilian pattern of injuries in armed conflicts – a systematic review

**DOI:** 10.1186/s13049-024-01299-7

**Published:** 2024-12-04

**Authors:** Karl Chevalley, Jonas Zimmerman, Anton Mittendorf, Filippa Sennersten, Anton Dalman, Safora Frogh, Theo Ringart, Yohan Robinson, Göran Sandström

**Affiliations:** 1 Department of Anesthesiology and Intensive Care, Institute of Clinical Sciences, Sahlgrenska Academy, University of Gothenburg, Gothenburg, Sweden; 2 Helicopter Emergency Medical Service, Region Västra Götaland, Gothenburg, Sweden; 3 Centre for Disaster Medicine, University of Gothenburg, Gothenburg, Sweden; 4 Swedish National Board of Forensic Medicine, Stockholm, Sweden; 5 Karolinska Institutet, Stockholm, Sweden; 6 Linköping University, Linköping, Sweden; 7 University of Uppsala, Uppsala, Sweden; 8 Department of War Studies, Swedish Defence University, Stockholm, Sweden

**Keywords:** War injuries, Armed conflict, Wounded, Civilian, Injury pattern, Non-combatant

## Abstract

**Background:**

War causes severe suffering and harm to the civilian population. Knowledge about civilian injury patterns constitutes a part of the dimensioned planning and preparedness for medical care and civilian defence in times of war. This systematic review is conducted on request from The Swedish National Board of Health and Welfare and includes civilian injury patterns in modern war.

**Methods:**

The aim of the study is to describe civilian injury patterns in war 1973–2023. We have conducted a systematic review using the Preferred Reporting Items for Systematic Reviews and Meta-Analyses (PRISMA) protocol. The protocol has been registered 2023-05-06 in PROSPERO (CRD4202321483).

**Results:**

The search resulted in 3455 identified articles. 1226 of those were duplicates. 2229 studies were assessed, and 1817 papers were excluded. 412 papers went through full text assessment resulting in 63 remaining papers. Injuries to the extremities constitutes 50%, followed by head injuries (26%) and injuries to the chest (18%). Notably, 23% of the wounded are children.

**Discussion:**

There is no standardized classification or method to report and describe civilian war injuries and the injury panorama. Variations in how the injuries were reported made synthesis of the results difficult. In the present survey we haven’t investigated mortalities and causes of death. Reliable data from recent wars, such as the ongoing war in Ukraine and Gaza, was missing from the open literature.

**Conclusions:**

The distribution of injuries seems comparable with data from World War II and the conflict in Korea. There is no standardized simple protocol to report civilian injuries in war. Ideally, a protocol should include even the severity impact of the injuries. Knowledge of civilian injury pattern and estimate of the total number of wounded is important to plan the civilian health care capabilities in war time.

## Introduction

War has devastating consequences for the civilian population. The civilian population is generally more vulnerable as, they are not trained to handle the complex environment that a war entails, nor do they have the protective equipment that soldiers in organized regular units have, i.e. body armour and helmets [1, 2]. The medical organization within the armed forces is also better trained and equipped to act within the chaotic and dangerous environment. The war in Ukraine and the recent years’ deterioration in the security situation has led Sweden, and many other countries in Europe, to decide to rebuild its civil national defence. Large scale combat operations (LSCO) involve combat activities that also effect civilian areas and results in huge numbers of casualties and wounded, military as well as civilian which can overwhelm the available medical treatment facilities (MTF). LSCO will result in wounded on a massive scale over time, not seen since World War II [3, 4]. Planning needs to be prepared in peacetime and is a responsibility for both civilian and military defence authorities.

During the Cold War, Sweden strategically prepared for conflict by pre-storing equipment and ensuring the capacity to rapidly repurpose many of the nation’s resources for civilian or military defence. The Swedish total defence concept involved most adult citizens (age 16-70y), who were assigned specific roles in support of national defence efforts [5, 6].

Military casualties and patterns of injury are frequently published in the literature [7]. As for the military personnel, the outcome in number of injured and their injuries are often reported according to standardized methods [8, 9]. This favours experience-based and scientific modifications of treatment methods and tactics [10]. To make preparation for wartime health-care capabilities there is a need for dimensioned planning conditions. Civilian patterns of injury are one of those requirements together with expected total number of injured civilians, military personnel, allied military personnel, volunteers, and enemies. Civilian patterns of injury are also important for planning of equipment and medical supplies in preparedness storages, and how to focus training courses on casualty combat care, war surgery and war time postoperative care. Such data has been published by the authorities such as The Swedish Defence Medical Board (reorganized 1994) together with The Swedish National Board of Health and Welfare. In 1994 they published “War surgical treatment principles”, in which historical data on injury patterns in war were reported [11]. This study aims to define an updated injury pattern for the civilian population in areas of modern armed conflict based on a systematic literature review.

## Methods

### Design

This is a systematic literature review on civilian injury patterns in modern wars between 1973 and 2023. The methodology follows the Preferred Reporting Items for Systematic Reviews and Meta-Analyses (PRISMA) guidelines [12], and the protocol was prospectively registered on May 6, 2023, in PROSPERO (CRD42023421483).

### Context

The Swedish National Board of Health and Welfare has assigned the Centre for Disaster Medicine at the University of Gothenburg to gather data regarding the civilian injury pattern in war to get an update of these figures (no. 15009/2023). Since the purpose is to provide a basis for healthcare planning in the Swedish total defence system, modern wars with contemporary warfare methods are particularly relevant. Therefore, only studies with data related to wars between January 1, 1973, and December 31, 2023, were included. The study was not limited geographically and encompasses conflicts worldwide. To allow a publicly releasable report, chemical, biological, and radiological (CBRN) warfare was excluded from the review.

### Publication types

Original articles, including cross-sectional, cohort, case-control, and randomized controlled studies, were included. Qualitative studies, such as interview or focus group studies, was also considered if they contained relevant data for the study. Only articles in Swedish or English were included. To avoid missing data, conference abstracts and theses were also considered for inclusion, in addition to peer-reviewed literature.

### Selection method

According to the PRISMA protocol the review-process followed defined phases.

The process of selecting studies for the systematic review included the following phases: title screening (exclusion by title), abstract screening (exclusion after abstract), inclusion (after full text), and data extraction.

The selection was carried out by all co-authors and each article has been assessed by at least two independent reviewers, and any disagreements between the reviewers have been resolved through discussion and consensus or through consultation with a third reviewer. Finally, the authors thoroughly reviewed each eligible article by the authors and the data, including the year of publication, author’s name(s), the title of the study, and its scope was registered. The scientific evidence of each selected article was then assessed and the wanted data was extracted. The variables for which data that was extracted are listed in Table 1.

**Table 1:** Civilian injury pattern in war 1973–2023

Years	1973–2023		1990–2015	1991–2021	1991–2000	2011–2019	1973–2021
Conflicts	All		Iraq and Afghanistan	Israel- Palestine	Former Yugoslavia	Syria	Other
Population wounded	158,811		50,570	8,624	71,805	10,971	17,095
**Injured body region**	**Percentage**	**Number** (total injuries^*^)					
Cranium Brain	26%	27,399 (104,585)	26%	14%	29%	29%	10%
Face	13%	3,041 (22,673)	15%	43%	9%	11%	3%
Neck	1%	135 (9,114)	4%	2%	3%	1%	1%
Chest	18%	5,770 (3, 822)	19%	42%	14%	25%	7%
Abdomen Pelvis	10%	10,018 (99,031)	20%	10%	9%	14%	3%
Spine	4%	428 (9,999)	N/A	5%	N/A	5%	4%
Upper extremities	32%	27,464 (86,787)	24%	45%	19%	29%	37%
Lower extremities	18%	16,249 (90,417)	51%	22%	10%	51%	33%
Burns	16%	1,131 (22,761)	3%	9%	N/A	11%	1%
Children	23%	31,652 (140,645)	28%	23%	20%	25%	18%

^*^Numbers within brackets represent total number of injuries documented in the included studies where the corresponding injury pattern occurs. The total sum varies because different studies presented different injury patterns

After the search was conducted, the results were imported into Endnote software (version 21) to remove duplicates. The results were then transferred to the Rayyan software [13] where nine reviewers included and excluded articles. Quantitative data were extracted from all included articles.

We used the Swedish translation of the Joanna Briggs Institute’s risk of bias tool for case series to assess the risk of bias in individual included studies. The overall evidence is presented in a risk-of-bias plot [14] (Table 2).

**Table 2:** JBI Risk of bias tool for case series

Data	Definition	Process
**Bibliographic information**	Author names, publication year, title, publication	Extracted by librarian.
**Conflict**	Geographic conflict area examined (e.g., Afghanistan)	Manually extracted by reviewers.
**Year**	Year of data collection	Manually extracted by reviewers.
**Study design**	Study design (randomized controlled trials, non-randomized controlled trials, cohort studies, case-control studies, and cross-sectional studies)	Manually extracted by reviewers.
**Population**	Total number of injured included in the study	Manually extracted by reviewers.
**Injuries by body part**	Number and proportion (%) of injuries by body part reported for the following body parts/areas: • Skull (including traumatic brain injury) • Face (including teeth) • Neck • Chest • Abdomen, pelvis, urogenital • Spine • Upper extremities (partially including lower) • Lower extremities • Burn injuries • Acute psychological reactions	Manually extracted by reviewers. If proportions (%) are provided, conversion to numbers will be performed. If numbers are provided, conversion to proportions (%) of the studied population (n total) will be performed.
**Child injuries**	Percentage (%) and number of injured children included in the study	Manually extracted by reviewers.
**Infections**	Number and proportion (%) of secondary infections after injury	Manually extracted by reviewers.
**Risk of bias**	According to JBI Risk of bias tool for case series	Manually extracted by reviewers.

The initially designed electronic search model used MEDLINE (1946-), Scopus (1900-), Embase (1947-) and NATO STO reports (2000-) and Web of Science to create a list of available literature in English, using the following search string:

*(“wounds and injuries“[MeSH Terms] OR (“wounds“[All Fields] AND “injuries“[All Fields]) OR “wounds and injuries“[All Fields] OR “trauma“[MeSH Terms] OR “trauma“[All Fields] OR “injury“[All Fields] OR “injuries“[All Fields]) AND (“civilians“[MeSH Terms] OR “civilian“[All Fields] OR “non-combatant“[All Fields] OR “noncombatant“[All Fields] OR “non-combat“[All Fields] OR “noncombat“[All Fields]) AND (“Armed conflict“[All Fields] OR “War“[All Fields])*

### Inclusion and exclusion criteria

Inclusions criteria: Original publications and reviews dated 1 January 1973 to 31 December 2023.

#### Exclusions criteria

Proceedings, editorials, news, non-conference abstracts, and non-relevant papers.

#### Ethical approval

This study complied with the ethical principles stipulated by Swedish law.

This study did not involve any human material or data regarding individuals, such as race, ethnic heritage, political views, religion, sexual habits, and health or physical interventions and was based on available published data in scientific sources, therefore there was no need for ethical approval.

## Results

**Fig. 1:**
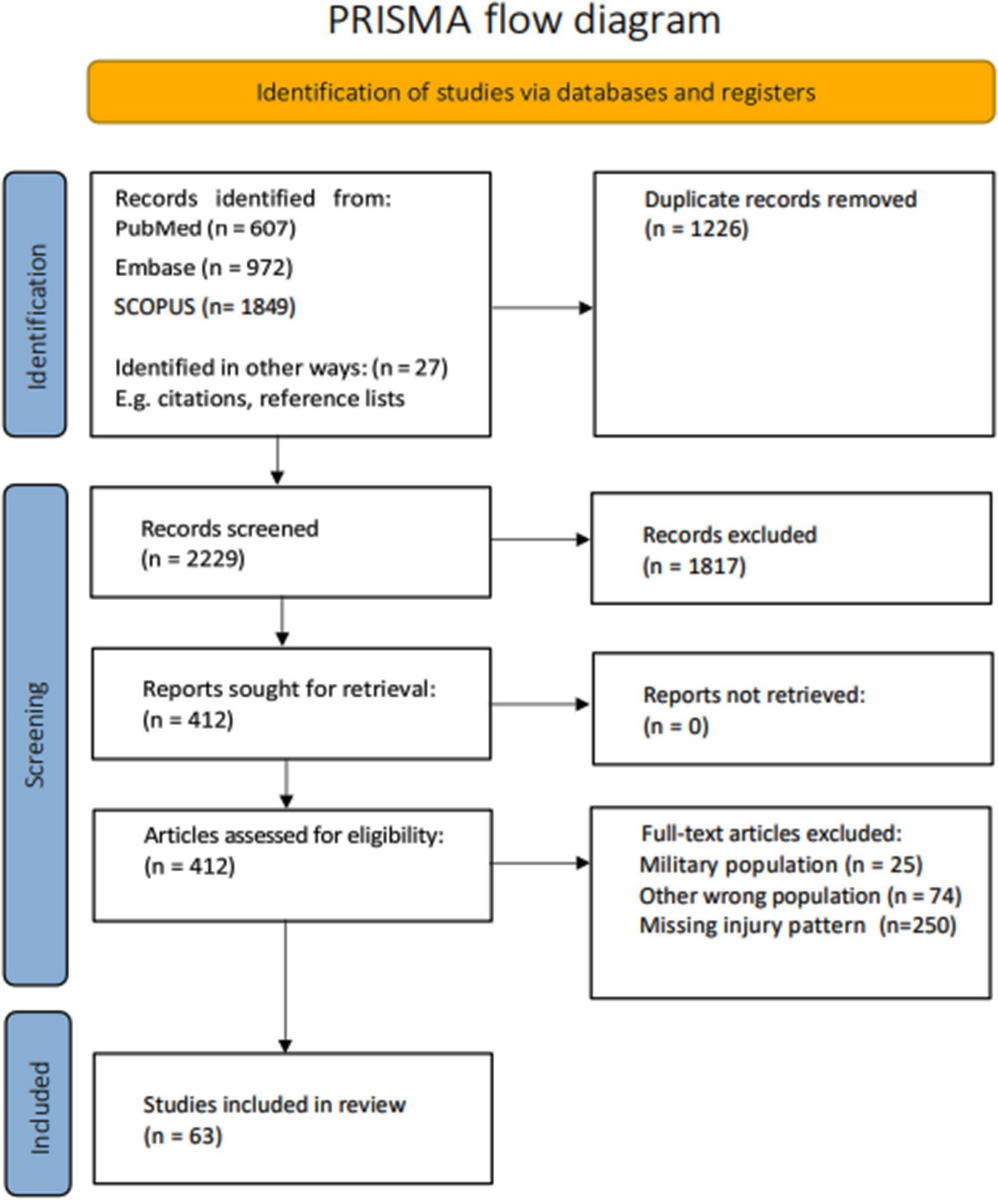
PRISMA diagram of inclusion studies

In this systematic review on civilian patterns of injury in war after 1973 we have categorized the injuries according to anatomic distribution. The initial search resulted in 3455 identified articles. 1226 of those were duplicates and therefore were not relevant for further evaluation. 2229 studies were assessed regarding title and abstract and 1817 papers were excluded. 412 papers went through full text assessment resulting in 67 remaining papers. Reasons for exclusion were military population (*n* = 25), wrong population of other reasons (*n* = 74) and no presentation of injury pattern (*n* = 250). Of those articles, 4 were double-publication and they were therefore also excluded. Finally, 63 papers remained for data-extraction (Fig. 1). No additional publications were included from the grey literature. All included studies were either case series (*n* = 62) or meta-analyses of case series (*n* = 1). Therefore, the level of evidence is limited.

The overall results of the literature search and review are presented in Table 1. The table is designed to resemble previous presentations of Swedish National Defence Planning [11]. The injuries reported in the articles have been summarized and categorized according to injury patterns. In addition, injury patterns have been reported separately for the four major conflict areas: Iraq and Afghanistan, Israel-Palestine, former Yugoslavia, Syria and others (Ireland, Lebanon, Eritrea, Democratic Republic of Congo, Libya, Cambodia, Sri Lanka, Iran, Somalia, and Pakistan).

According to our findings the most frequent injuries are those to the extremities which constitutes 50% followed by head injuries (26%) and injuries to the chest (18%). Among civilian wounded in this material, 23% are children. The total percentage injuries are more the 100% because some of the wounded sustained injuries to more than one body region. It appears that the distribution of some injury patterns is similar in different conflict areas, but for some types of injuries there is a wider variability. Injuries to the cranium/brain affected 26–29% of the wounded in the wars in Iraq-Afghanistan, former Yugoslavia and Syria. However, injuries to the lower extremities varied between 10 and 51% among the different conflict areas. These differences have not been analysed further.

## Discussion

The purpose of this study was to identify civilian injury patterns in modern warfare between 1973 and 2023 through a systematic literature review. We have found that injuries in upper and lower extremities were most frequently reported and constituted about 50% of the wounded (32% and 18% respectively). Thorax, abdominal and spinal injuries are less commonly reported in the literature. However, these types of injuries are probably more common among those who didn’t survive and are therefore reported as deceased instead of wounded [2]. In the present survey we haven’t investigated mortalities and causes of death. Survival bias is important to consider for interpretation of the findings and for future wartime planning regarding on scene casualty care, treatment, transportation and the role of additional MTF [3, 4].

Children are particularly vulnerable in a war situation [15, 16]. Almost a fourth of the civilian wounded were children in this literature survey. However, the distribution of injury pattern is unknown. In addition, the age distribution is not defined.

The injury panorama in war could be generated by different types of mechanisms and cause a variety of blast-, penetrating-, fire- and crush injuries and reflects the clinical reality of war.

There is no standardized classification or method to report and describe civilian war injuries and the injury panorama. We observed that there was no uniform reporting of injury distribution in the included studies. Other authors have come to the same conclusion [2]. Variations in how the injuries were reported made synthesis of the results difficult. Some studies focused only on the most severely injuries, while others included all injuries. Some studies chose to report combined extremities instead of distinguishing between upper and lower extremities. This difference in reporting has probably affected our presented synopsis of the injury panorama. Some studies were excluded because civilian and military casualties were mixed, which limited the basis for analysis and the ability to draw conclusions about injury outcomes for both groups. As far as we noticed there is not a common standardized method in how the severity of the injuries are reported. There are approaches to standardized registration of injury data, such as the US Department of Defense Trauma Register and the ICRC (International Committee of Red Cross) Casualty Register. There are probably many challenges and potential sources of bias in collecting and compiling data of civilian wounded in wartime. Wounded can be distributed to different MTF, some patients might have been referred or refused upon arrival before registration. There is also a possibility that not all wounded patients were registered correctly and that the information from health services and hospitals stressed by conflicts and war might not be completely accurate or reliable.

Finally, it can be noted that reliable data from recent wars, such as the ongoing war in Ukraine and Gaza, was missing from the open literature.

### Limitations

The risk of publication bias, the selective publication of research studies based on their results might impact the result.

Survival bias, results concentrating on persons that have survived while we have no adequate information about those who did not.

Selection bias, a proper randomisation has not been possible to perform. We cannot know if the wounded cohort is representative for the whole population within the areas studied.

Language bias, a systematic bias due to the selection of reports published. All the publications used in this review are in English. Consequently, some interesting data in other languages might be missing.

## Conclusions

“War is the realm of uncertainty; three quarters of the factors on which action in war is based are wrapped in a fog of greater or lesser uncertainty” according to Carl von Clausewitz, a renowned military strategist [17]. Despite the uncertainties which associates with data collection of civilian injury patterns in war, this systematic review indicate that the civilian injury pattern is relatively consistent over time. The distribution of injuries is comparable with data from World War II and the conflict in Korea [11]. There is no standardized simple protocol to report civilian injuries in war. Ideally, a protocol should include even the severity impact of the injuries. If a standardized protocol could be agreed on and used, it would facilitate scientific studies and planning of medical logistics before and during an armed conflict. Strategic decisions for national and allied defence planning must be based on the highest level of scientific evidence to rebuild preparedness for the health care system. An updated understanding of civilian injury patterns is essential for determining the appropriate storage of medical supplies and for planning the necessary training and logistical preparations for healthcare personnel. The paediatric population among injured in armed conflict is still underreported and requires further study. In addition to knowledge of civilian and military injuries, there is a need for an estimate of the numbers of wounded civilian, military, allied and enemy casualties over time in a future conflict. These basic planning dimensions of the type and total numbers of wounded are fundamental to allocate the right resources for the combined wartime health care capabilities. Future studies are needed to include both wounded civilian and military personnel in LSCO such as the ongoing conflicts is Ukraine and Gaza. Additional future research needs to contribute to the development of combined civilian and military guidelines in LSCO, for example war time triage, treatment guidelines and war surgery. Wartime capabilities for combined medical resources require careful preplanning, ensuring that when mobilised, they can rapidly transition from a peacetime structure to a significantly expanded wartime operation. This involves the deployment of additional personnel, equipment, consumable materials, and bolstered logistics and reserves across all areas to meet the heightened demands of conflict [3, 4, 18]. Based on the available data and our own experience we recommend that the total defence healthcare system is evaluated continuously during regular exercises to fine-tune the plans and preparations to meet civilian and military defence baseline requirements [3, 4, 18, 19].

## Appendix 1 Included articles the review

**Table:** 

No	Year	Authors	Title	Journal
1	2013	Karakuş, A.,et al.	The reflection of the Syrian civil war on the emergency department and assessment of hospital costs.	Ulusal Travma ve Acil Cerrahi Dergisi
2	2017	Duzkoylu, Y.,et al.	Physical Trauma among Refugees: Comparison between Refugees and Local Population Who Were Admitted to Emergency Department - Experience of a State Hospital in Syrian Border District.	Journal of Environmental and Public Health
3	2017	Er, E., Ş. K., et al.	Analyses of demographical and injury characteristics of adult and paediatric patients injured in Syrian civil war.	American Journal of Emergency Medicine
4	2017	Kocamer Şimşek, B., M., et al.	Characteristics of the injuries of Syrian refugees sustained during the civil war.	Ulusal Travma ve Acil Cerrahi Dergisi
5	2017	Qasaimeh, G., et al.	The pattern of the Syrian refugee’s injuries managed in King Abdullah University Hospital (Jordan).	Eur J Trauma Emerg Surg
6	2018	Kuvandik, G., et al.	Epidemiology and cost of burns in emergency department during Syrian civil war.	Bratisl Lek Listy
7	2020	McIntyre, J.	Syrian Civil War: a systematic review of trauma casualty epidemiology.	BMJ Mil Health
8	2021	Cakmak, F., et al.	Distribution and cost of Syrian refugees operated on in Southeastern Anatolia, Turkey.	Eastern Mediterranean Health Journal
9	2021	Tabakan, I., et al.	Reconstruction of firearm and blast injuries in Syrian war refugees.	International Journal of Clinical Practice
10	1993	Bajec, J., et al.	Post Gulf war explosive injuries in liberated Kuwait.	Injury
11	1993	Korver, A. J. H.	Amputees in a hospital of the International Committee of the Red Cross.	Injury
12	1994	Korver, A. J. H.	Outcome of war-injured patients treated at first aid posts of the International Committee of the Red Cross.	Injury
13	1999	Michael, M., et al.	Incidence of weapon injuries not related to interfactional combat in Afghanistan in 1996: prospective cohort study.	BMJ
14	2003	Bilukha, O. O., et al.	Death and Injury from Landmines and Unexploded Ordnance in Afghanistan.	JAMA
15	2008	Bilukha, O. O., et al.	The lasting legacy of war: epidemiology of injuries from landmines and unexploded ordnance in Afghanistan, 2002–2006.	Prehosp Disaster Med
16	2010	Donaldson, R. I., et al.	Injury burden during an insurgency: the untold trauma of infrastructure breakdown in Baghdad, Iraq.	J Trauma
17	2010	Edwards, M. J., et al.	Blast injury in children: an analysis from Afghanistan and Iraq, 2002–2010.	J Trauma Acute Care Surg
18	2017	Hemat, H., et al.	Before the bombing: High burden of traumatic injuries in Kunduz Trauma Center, Kunduz, Afghanistan.	PLoS ONE
19	2019	Schauer, S. G., et al.	Analysis of Injuries and Prehospital Interventions Sustained by Females in the Iraq and Afghanistan Combat Zones.	Prehosp Emerg Care
20	2022	Maitland, L., et al.	Analysis of 983 civilian blast and ballistic casualties and the generation of a template of injury burden: An observational study.	eClinicalMedicine
21	1991	Karsenty, E., et al.	Medical aspects of the Iraqi missile attack on Israel.	Israel Journal of Medical Sciences
22	2010	Peleg, K. and D. H. Jaffe	“Are injuries from terror and war similar? A comparison study of civilians and soldiers.	Ann Surg
23	2011	Adini, B., et al.	Do modern conflicts create different medical needs?	American Journal of Emergency Medicine
24	2017	Ellenberg, E., et al.	Lessons From Analyzing the Medical Costs of Civilian Terror Victims: Planning Resources Allocation for a New Era of Confrontations.	Milbank Q
25	2018	Mosleh, M., et al.	The burden of war-injury in the Palestinian health care sector in Gaza Strip.	BMC Int Health Hum Rights
26	2021	Iordache, S. D., et al.	Treatment of Peripheral Nerve Injuries in Syria’s War Victims: Experience from a Northern Israeli Hospital.	Isr Med Assoc J
27	1994	Bogdanovic, S., et al.	Dubrovnik General Hospital: Civilian surgery in the besieged town.	Croatian Medical Journal
28	1994	Pretto, E. A., et al.	Emergency medical services during the siege of Sarajevo, Bosnia and Herzegovina: a preliminary report.	Prehosp Disaster Med
29	1995	VanRooyen, M. J., et al.	The incidence and outcome of penetrating and blunt trauma in central Bosnia: The Nova Bila Hospital for war wounded.	Journal of Trauma - Injury, Infection and Critical Care
30	1998	Rukavina, A.	War injuries in civilians treated in Pozega Hospital, Croatia.	J R Army Med Corps
31	2002	Suljević, I. and I. Surković	Medical aspects of the mass-scale civilian casualties at Sarajevo Markale market on August 28, 1995: triage, resuscitation, and treatment.	Croat Med J
32	2003	Kinra, S. and M. E. Black	Landmine related injuries in children of Bosnia and Herzegovina 1991–2000: Comparisons with adults.	Journal of Epidemiology and Community Health
33	1973	Haddad, F. S.	Nature and management of penetrating head injuries during the civil war in Lebanon.	Canadian Journal of Surgery
34	2000	Hanevik, K. and G. Kvale	Landmine injuries in Eritrea.	British Medical Journal
35	2000	Meade, P. and J. Mirocha	Civilian landmine injuries in Sri Lanka.	Journal of Trauma - Injury, Infection and Critical Care
36	2008	Soroush, A., F. et al.	Amputations due to landmine and unexploded ordinances in post-war Iran.	Arch Iran Med
37	2009	Bendinelli, C.	Effects of land mines and unexploded ordnance on the pediatric population and comparison with adults in rural Cambodia.	World J Surg
38	2012	Mohamadzadeh, H., et al.	Landmine victims in Iran Kurdistan; demographic features and accident characteristics.	Pakistan Journal of Medical Sciences
39	2013	Bodalal, Z. and S. Mansor	Gunshot injuries in Benghazi-Libya in 2011: the Libyan conflict and beyond.	Surgeon
40	2017	Napier, R. J., et al.	An imperfect peace: Trends in paramilitary related violence 20 years after the Northern Ireland ceasefires.	Ulster Medical Journal
41	2021	Budema, P. M., et al.	Fatal and nonfatal firearm injuries in the eastern Democratic Republic of Congo: a hospital-based retrospective descriptive cohort study assessing correlates of adult mortality.	BMC Emergency Med
42	2021	Muhrbeck, M., et al.	Predicting surgical resource consumption and in-hospital mortality in resource-scarce conflict settings: a retrospective study.	BMC Emerg Med
43	2022	Sharma, P., A. Sharma and K. Rao	The changing paradigm of injuries and their outcome in an international conflict zone.	Journal of Marine Medical Society
44	2023	Mohamed, A. Y., et al.	Epidemiological characteristics and comparative outcome of blast versus gunshot injuries of the extremities in Somalia.	Journal of Orthopaedic Surgery and Research
45	2001	Terzić, J., et al.	Children war casualties during the 1991–1995 wars in Croatia and Bosnia and Herzegovina.	Croat Med J
46	2008	Spinella, P. C., et al.	Pediatric trauma in an austere combat environment.	Crit Care Med
47	2013	Wilson, K. L., et al.	Pediatric trauma experience in a combat support hospital in eastern Afghanistan over 10 months, 2010 to 2011.	Am Surg
48	2012	Borgman, M., et al.	Isolated pediatric burn injury in Iraq and Afghanistan.	Crit Care Med
49	2012	Edwards, M. J., et al.	Blast injury in children: an analysis from Afghanistan and Iraq, 2002–2010.	J Trauma Acute Care Surg
50	2014	McKechnie, P. S., et al.	Pediatric surgery skill sets in role 3: The Afghanistan experience.	Military Medicine
51	2014	Villamaria, C. Y., et al.	Wartime vascular injuries in the pediatric population of Iraq and Afghanistan: 2002–2011.	J Pediatr Surg
52	2015	Hemmati, M. A., et al.	Mental health disorders in child and adolescent survivors of post-war landmine explosions.	Mil Med Res
53	2015	Pannell, D., et al.	Factors affecting mortality of pediatric trauma patients encountered in Kandahar, Afghanistan.	Canadian Journal of Surgery
54	2015	Sokol, K. K., et al.	Prehospital interventions in severely injured pediatric patients: Rethinking the ABCs.	J Trauma Acute Care Surg
55	2019	Gale, H. L., et al.	An Analysis of Outcomes and Interventions for Female Pediatric Casualties in Iraq and Afghanistan.	Military Medicine
56	2020	Marenco, C. W., et al.	Validation of shock index pediatric adjusted for children injured in warzones.	Journal of Trauma and Acute Care Surgery
57	2020	Naaman, O., et al.	Syria civil war pediatric casualties treated at a single medical centre.	Journal of Pediatric Surgery
58	2020	Schauer, et alS. G.,	An analysis of the pediatric casualties undergoing massive transfusion in Iraq and Afghanistan.	Am J Emerg Med
59	2020	Thompson, D. C., et al.	The pattern of paediatric blast injury in Afghanistan.	BMJ Military Health
60	2021	Cuenca, C. M., et al.	Incidence of post-traumatic seizures in children during combat operations in Afghanistan and Iraq.	Injury
61	2022	Korkmaz, I., et al.	Imaging in paediatric blast injuries: musculoskeletal injuries in the Syrian Civil War.	Clin Radiol
62	2021	Marenco, C. W., et al.	Validation of shock index pediatric adjusted for children injured in warzones.	Journal of Trauma and Acute Care Surgery
63	2023	Ramaraj, P.; et al.	Epidemiology of traumatically injured Yemeni civilians treated at the Omani National Trauma Centre over a 2-year period: a retrospective cohort study	BMJ Mil Health

## Data Availability

Preferred Reporting Items for Systematic Reviews and Meta-Analyses (PRISMA) protocol has been registered 2023-05-06 in PROSPERO (CRD4202321483). All articles screened are available in a Rayyan software library and can be shared upon request. All included articles are reported in an appendix.
